# Supplementary material for: Respiratory frequency-tunable dynamic imaging for lung function: New exam method using chest X-ray cine imaging considering various respiratory diseases
Source: PLoS One. 2022 Nov 17;17(11):e0276859. doi: 10.1371/journal.pone.0276859 (PMC9671319; doi:10.1371/journal.pone.0276859)

No 1

スパイログラム	測定値	予測値	%予測値
肺活量　VC (L)	3.67	2.99	122.7
予備呼気量　ERV (L)	0.96	1.24	77.4
予備吸気量　IRV (L)	1.34		
努力性肺活量　FVC (L)	3.63	2.99	121.4
一秒量　FEV1.0 (L)	1.92	1.85	103.8
一秒率（G）FEV1.0%(%)	52.89	65.68	80.5
一秒率（T）FEV1.0%(%)	52.32		
最大中間呼気流量 (L/s)	0.50	2.56	19.5
ATI エアートラッピング指数	1.09		
公害指数	66.2		
フローボリューム曲線			
ピークフロー (L/s)	4.86	8.52	57.0
V̇75 (L/s)	2.54	6.59	38.5
V̇50 (L/s)	0.74	4.00	18.5
V̇25 (L/s)	0.17	1.04	16.3
V̇50/V̇25	4.35		
V̇25/HT (L/s/m)	0.11	1.03	10.7
IV50 (L/s)	3.39		
V̇50/IV̇50	0.22		
IV̇50/V̇50	4.58		
肺気量			
機能的残気量　FRC (L)	3.32	3.28	101.2
全肺気量　TLC (L)	6.03	4.76	126.7
残気量　RV (L)	2.36	1.46	161.6
肺活量　VC (L)	3.67	2.99	122.7
残気率　RV/TLC (%)	39.14	40.68	96.2
肺拡散能力			
DLCO (mL/min/mmHg)	13.75	11.59	118.6
VA (STPD) (L)	4.80		
DLCO/VA (mL/min/mmHg/L)	2.86	4.28	66.8
B.H.TIME (s)	10.28		

コメント.

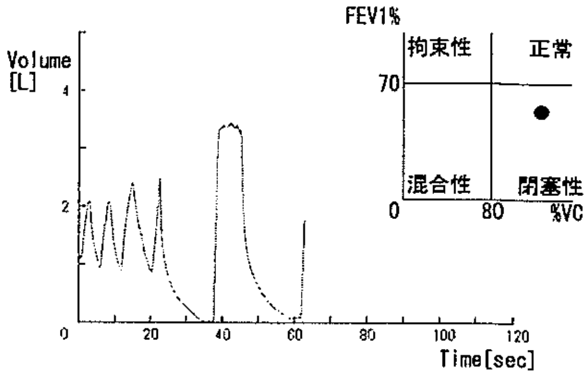

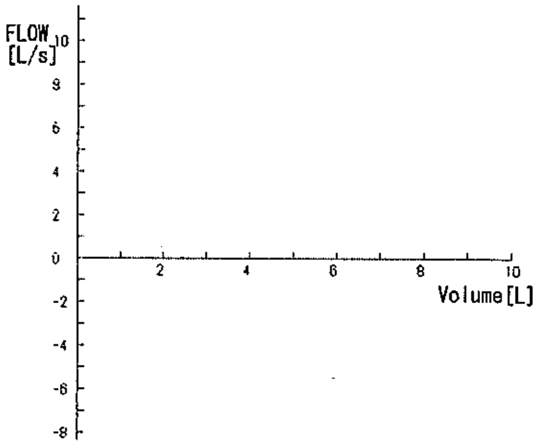

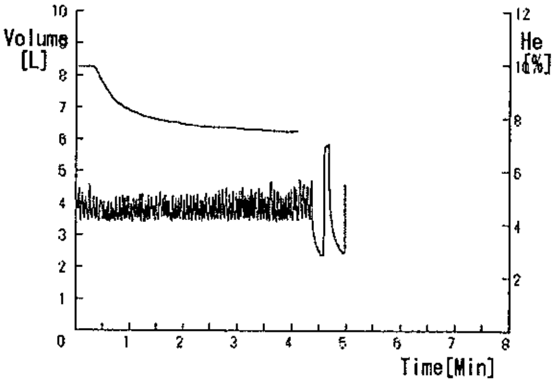

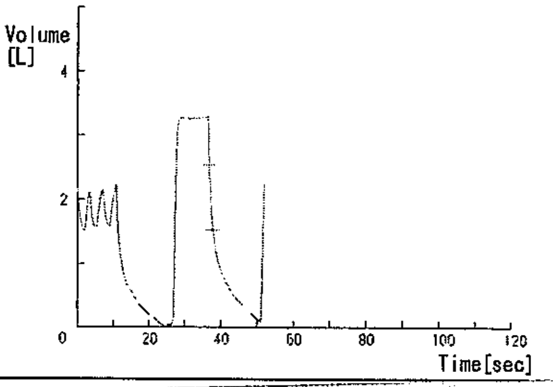

No1

FRC		測定値	予測値	%予測値
FRC	(L)	3.32	3.28	101.2
RV	(L)	2.36	1.46	161.6
TLC	(L)	6.03	4.76	126.7
RV/TLC		39.14	40.68	96.2
VC	(L)	3.67	2.99	122.7
IRV	(L)	1.81		
TV	(L)	0.90		
ERV	(L)	0.96		
[illegible]		[illegible]	[illegible]	[illegible]
DLCO	(mL/min/mmHg)	13.75	11.59	118.6
DLCO'	(mL/min/mmHg)	13.72	10.24	134.0
RV (STPD)	(L)	1.95		
IVC (STPD)	(L)	2.99		
VA (STPD)	(L)	4.80		
VA' (STPD)	(L)	4.79		
DLCO/VA	(mL/min/mmHg/L)	2.86	4.28	66.8
B. H. T.	(s)	10.28		
FIHe	(%)	9.70		
FAHe	(%)	5.81		
FICO	(%)	0.289		
FACO	(%)	0.122		

CV		測定値	予測値	%予測値
CV	(L)			
CC	(L)			
RV	(L)			
CV/VC	(%)			
CC/TLC	(%)			
⊿N2	(%)			
MSAP				
ADS	(L)			

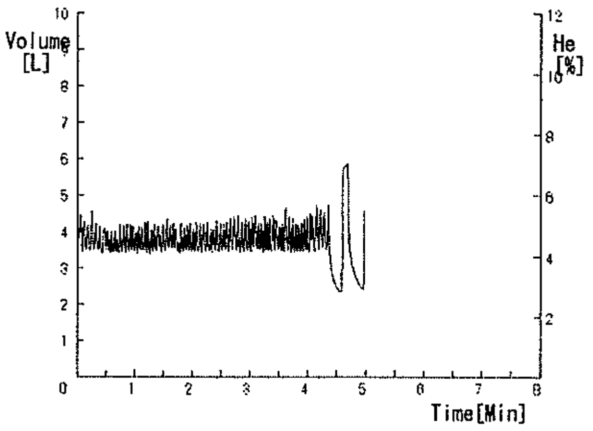

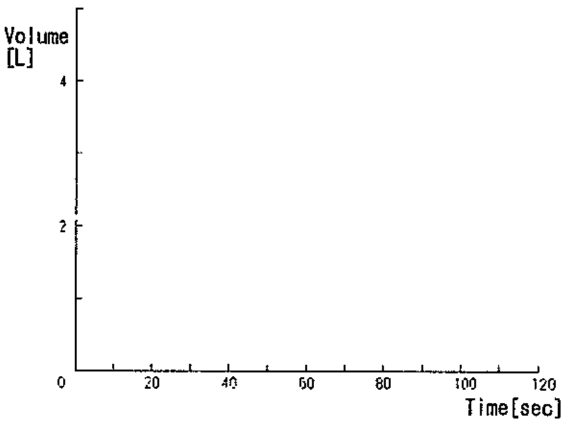

コメント.

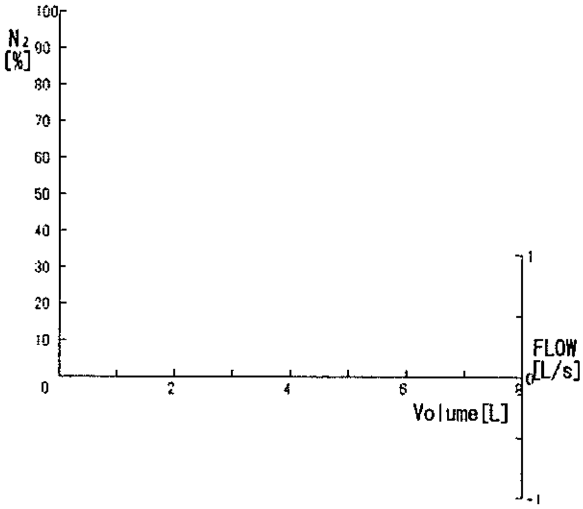

No1

[illegible]

No2

スパイログラム	測定値	予測値	%予測値
肺活量 VC (L)	3.89	3.32	117.2
予備呼気量 ERV (L)	1.37	1.34	102.2
予備吸気量 IRV (L)	1.22		
努力性肺活量 FVC (L)	3.85	3.32	116.0
一秒量 FEV1.0 (L)	2.54	2.43	104.5
一秒率（G） FEV1.0%(%)	65.97	65.68	100.4
一秒率（T） FEV1.0%(%)	65.30		
最大中間呼気流量 (L/s)	1.16	2.31	50.2
ATI エアートラッピング指数	1.03		
公害指数	84.9		
フローボリューム曲線			
ピークフロー (L/s)	7.32	8.96	81.7
V̇75 (L/s)	4.78	7.09	67.4
V̇50 (L/s)	1.43	4.28	33.4
V̇25 (L/s)	0.51	1.07	47.7
V̇50／V̇25	2.80		
V̇25／HT (L/s/m)	0.31	0.89	34.8
IV50 (L/s)	4.43		
V̇50／IV̇50	0.32		
IV̇50／V̇50	3.10		
肺気量			
機能的残気量 FRC (L)	4.19	3.97	105.5
全肺気量 TLC (L)	6.71	5.17	129.8
残気量 RV (L)	2.82	1.93	146.1
肺活量 VC (L)	3.89	3.32	117.2
残気率 RV／TLC (%)	42.03	43.43	96.8
肺拡散能力			
DLCO (mL/min/mmHg)	11.69	12.40	94.3
VA (STPD) (L)	5.34		
DLCO VA (mL/min/mmHg/L)	2.19	3.90	56.2
B.H.TIME (s)	10.65		

コメント.

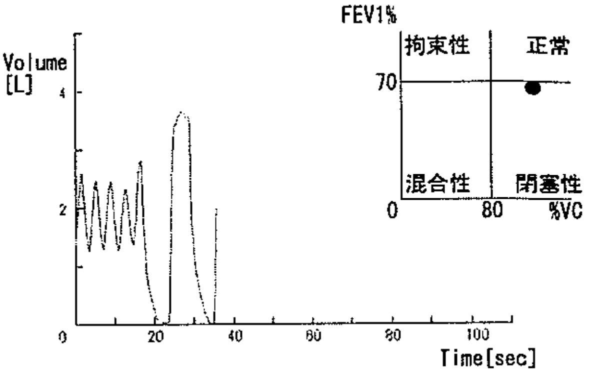

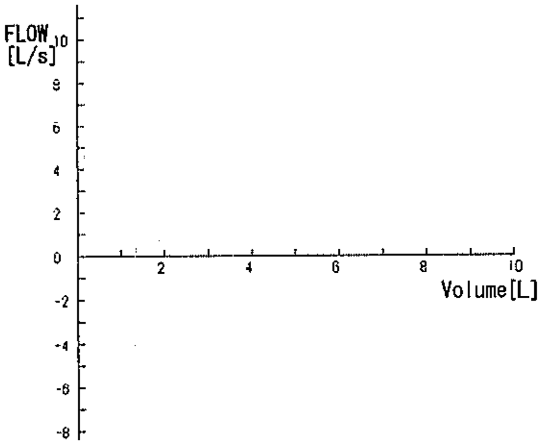

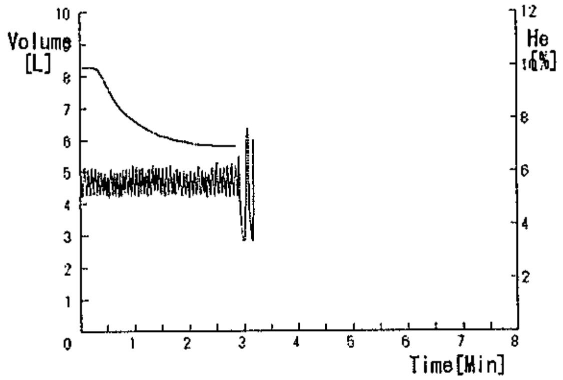

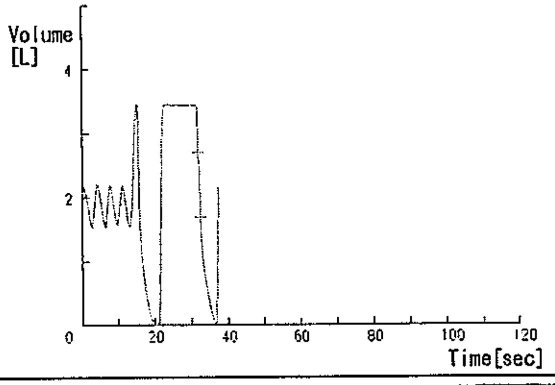

No 2

[illegible]

FRC		測定値	予測値	%予測値
FRC	(L)	4.19	3.97	105.5
RV	(L)	2.82	1.93	146.1
TLC	(L)	6.71	5.17	129.8
RV/TLC		42.03	43.43	96.8
VC	(L)	3.89	3.32	117.2
IRV	(L)	1.58		
TV	(L)	0.94		
ERV	(L)	1.37		
[illegible]		[illegible]	[illegible]	[illegible]
DLCO	(mL/min/mmHg)	11.69	12.40	94.3
DLCO'	(mL/min/mmHg)	12.46	12.60	98.9
RV (STPD)	(L)	2.33		
IVC (STPD)	(L)	3.15		
VA (STPD)	(L)	5.34		
VA' (STPD)	(L)	5.69		
DLCO/VA	(mL/min/mmHg/L)	2.19	3.90	56.2
B. H. T.	(s)	10.65		
FIHe	(%)	9.73		
FAHe	(%)	5.19		
FICO	(%)	0.277		
FACO	(%)	0.112		
CV		測定値	予測値	%予測値
CV	(L)			
CC	(L)			
RV	(L)			
CV/VC	(%)			
CC/TLC	(%)			
⊿N_2	(%)			
MSAP				
ADS	(L)			

No 2

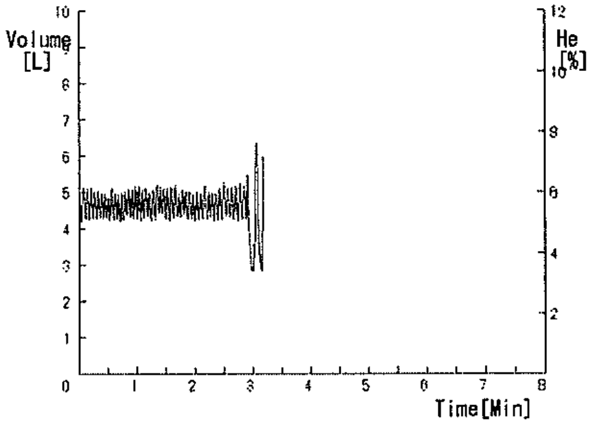

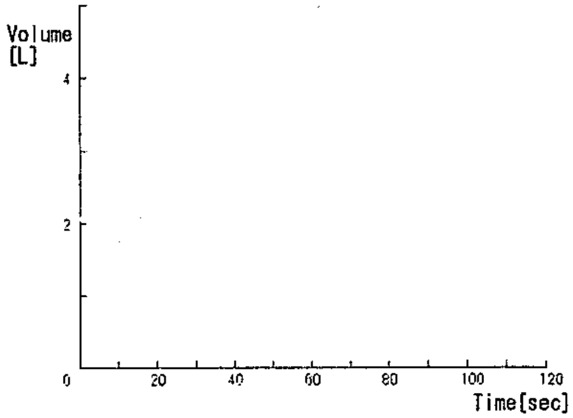

コメント.

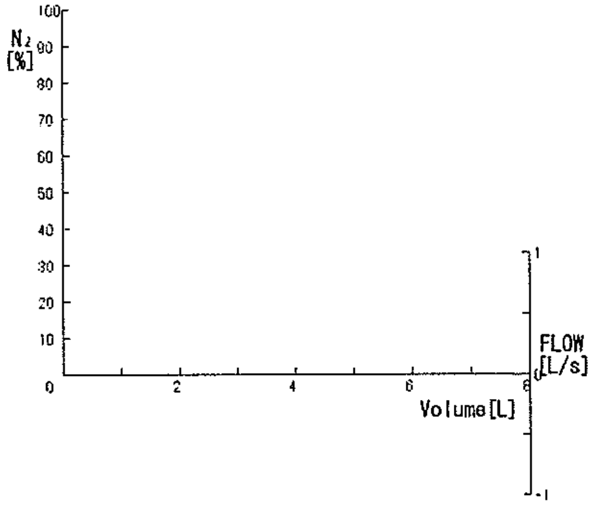

V23 No3

[illegible]

スパイログラム	測定値	予測値	%予測値
肺活量 VC (L)	3.56	3.30	107.9
予備呼気量 ERV (L)	0.81	1.37	59.1
予備吸気量 IRV (L)	1.83		
努力性肺活量 FVC (L)	3.37	3.30	102.1
一秒量 FEV1.0 (L)	2.22	2.39	92.9
一秒率（G） FEV1.0%(%)	65.88	65.68	100.3
一秒率（T） FEV1.0%(%)	62.36		
最大中間呼気流量 (L/s)	0.90	2.92	30.8
ATI エアートラッピング指数	5.34		
公害指数	68.9		
フローボリューム曲線			
ピークフロー (L/s)	7.63	8.93	85.4
V̇75 (L/s)	5.40	7.17	75.3
V̇50 (L/s)	1.49	4.43	33.6
V̇25 (L/s)	0.29	1.30	22.3
V̇50／V̇25	5.14		
V̇25／HT (L/s/m)	0.18	1.04	17.3
IV50 (L/s)	4.94		
V̇50／IV̇50	0.30		
IV̇50／V̇50	3.32		
肺気量			
機能的残気量 FRC (L)			
全肺気量 TLC (L)			
残気量 RV (L)			
肺活量 VC (L)			
残気率 RV／TLC (%)			
肺拡散能力			
DLCO (mL/min/mmHg)			
VA (STPD) (L)			
DLCO/VA (mL/min/mmHg/L)			
B.H.TIME (s)			

コメント.

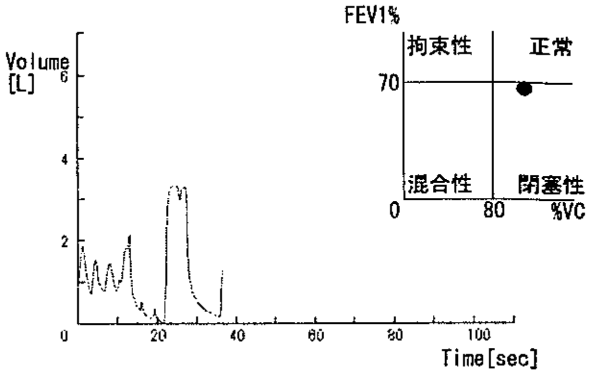

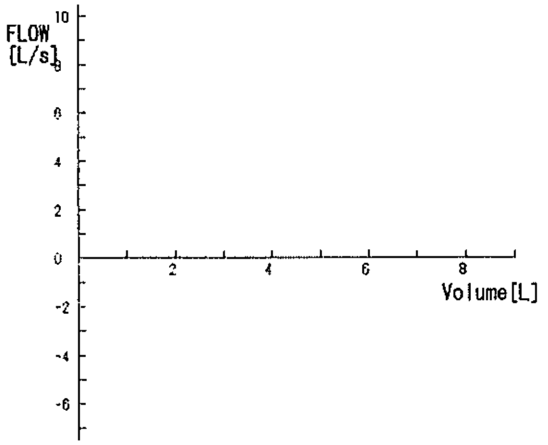

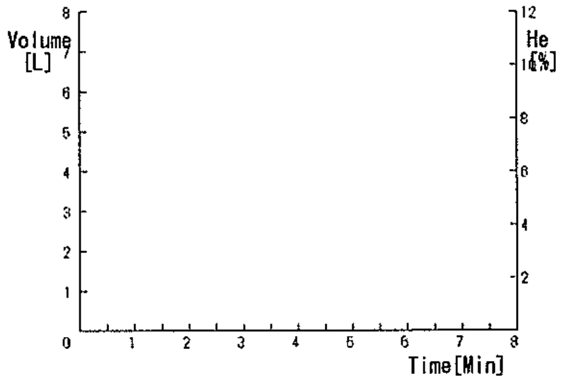

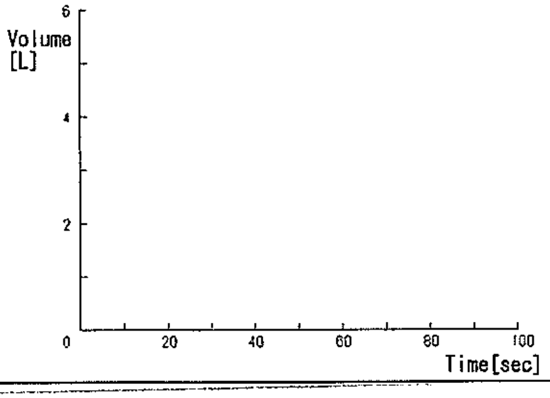

104

No4

[illegible]

スパイログラム	測定値	予測値	%予測値
肺活量 VC (L)	2.57	3.24	79.3
予備呼気量 ERV (L)	1.01	1.31	77.1
予備吸気量 IRV (L)	0.37		
努力性肺活量 FVC (L)	2.14	3.24	66.0
一秒量 FEV1.0 (L)	1.01	2.30	43.9
一秒率（G） FEV1.0%(%)	47.20	65.68	71.9
一秒率（T） FEV1.0%(%)	39.30		
最大中間呼気流量 (L/s)	0.38	2.59	14.7
ATI エアートラッピング指数	16.73		
公害指数	33.0		
フローボリューム曲線			
ピークフロー (L/s)	3.04	8.86	34.3
V̇75 (L/s)	0.84	6.95	12.1
V̇50 (L/s)	0.46	4.18	11.0
V̇25 (L/s)	0.18	1.01	17.8
V̇50/V̇25	2.56		
V̇25/HT (L/s/m)	0.11	0.97	11.3
IV50 (L/s)	2.23		
V̇50/IV̇50	0.21		
IV̇50/V̇50	4.85		
肺気量			
機能的残気量 FRC (L)			
全肺気量 TLC (L)			
残気量 RV (L)			
肺活量 VC (L)			
残気率 RV/TLC (%)			
肺拡散能力			
DLCO (mL/min/mmHg)			
VA (STPD) (L)			
DLCO/VA (mL/min/mmHg/L)			
B. H. TIME (s)			

コメント.

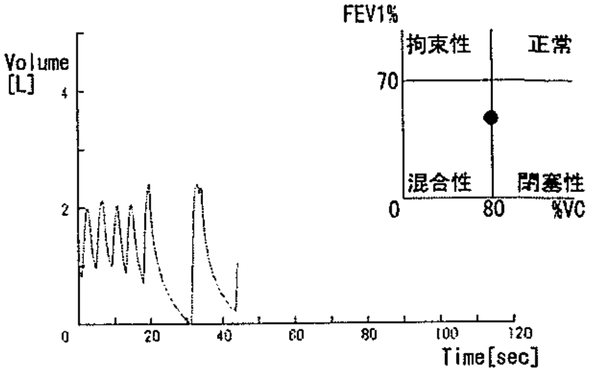

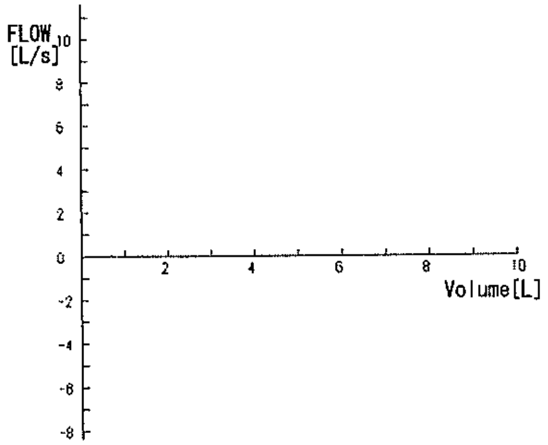

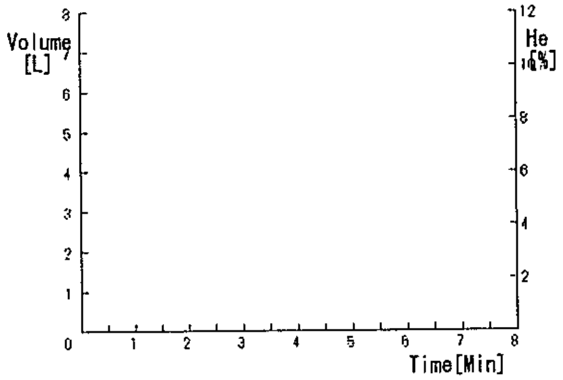

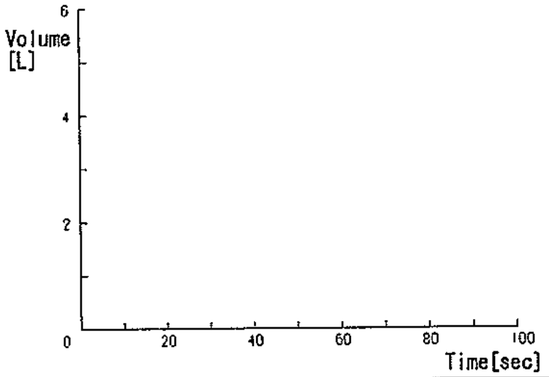

喫　煙：喫煙なし　(BI=　)　体表面積：[illegible]

No5

スパイログラム	測定値	予測値	%予測値
肺活量　VC (L)	2.55	3.38	75.4
予備呼気量　ERV (L)	1.16	1.39	83.5
予備吸気量　IRV (L)	0.84		
努力性肺活量　FVC (L)	2.37	3.38	70.1
一秒量　FEV1.0 (L)	0.90	2.54	35.4
一秒率(G)　FEV1.0%(%)	37.97	65.68	57.8
一秒率(T)　FEV1.0%(%)	35.29		
最大中間呼気流量 (L/s)	0.24	2.92	8.2
ATI　エアートラッピング指数	7.06		
公害指数	27.5		
フローボリューム曲線			
ピークフロー (L/s)	2.85	9.03	31.6
V̇75 (L/s)	0.74	7.28	10.2
V̇50 (L/s)	0.26	4.48	5.8
V̇25 (L/s)	0.16	1.28	12.5
V̇50/V̇25	1.63		
V̇25/HT (L/s/m)	0.09	1.02	8.8
IV50 (L/s)	2.48		
V̇50/IV̇50	0.10		
IV̇50/V̇50	9.54		
肺気量			
機能的残気量　FRC (L)	5.40	3.86	139.9
全肺気量　TLC (L)	6.79	5.39	126.0
残気量　RV (L)	4.24	1.85	229.2
肺活量　VC (L)	2.55	3.38	75.4
残気率　RV/TLC (%)	62.44	39.12	159.6
肺拡散能力			
DLCO (mL/min/mmHg)	15.46	16.04	96.4
VA (STPD) (L)	5.54		
DLCO/VA (mL/min/mmHg/L)	2.79	4.26	65.5
B.H.TIME (s)	12.85		

コメント.

薬効前、再検しましたが再現性とれず。

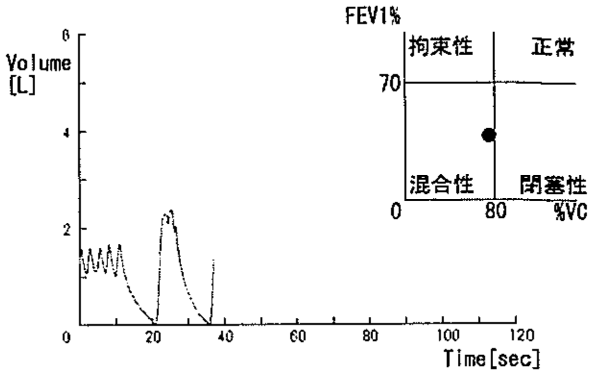

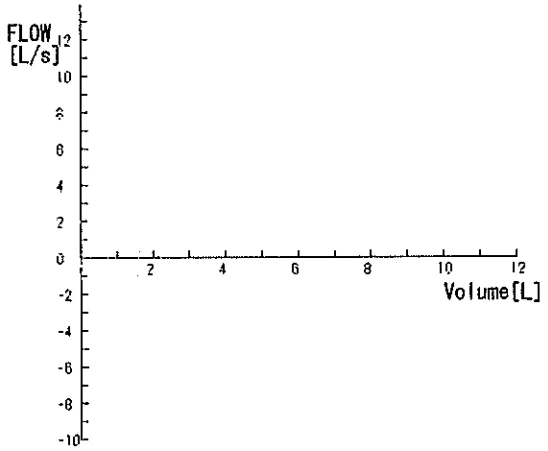

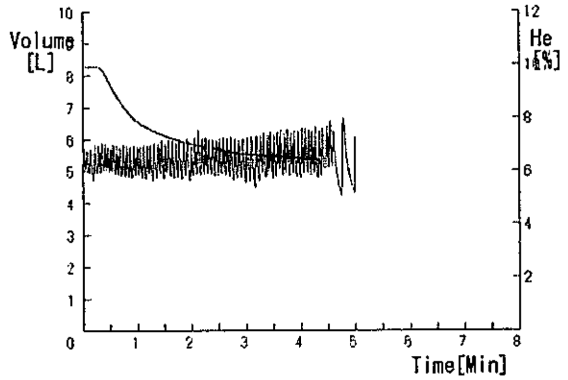

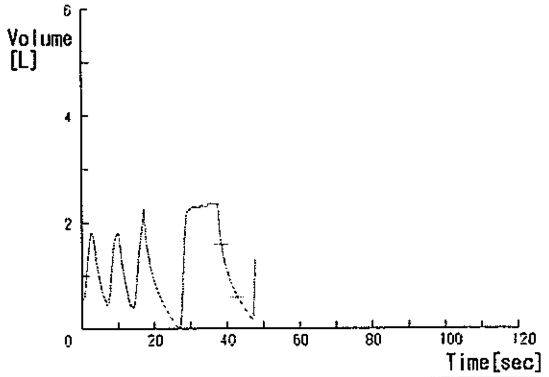

No5　　　　No5

喫　煙：喫煙なし　(BI=　)　体表面積：[illegible]

SVC	吸入前	吸入後	改善率
VC (L)	2.55	2.98	16.9
ERV (L)	1.16	0.60	-48.3
IRV (L)	0.84	0.67	-20.2
TV (L)	0.55	1.71	210.9
IC (L)	1.39	2.38	71.2
[illegible]	吸入前	[illegible]	[illegible]
FVC (L)	2.37	2.88	21.5
FEV1.0 (L)	0.90	0.96	6.7
FEV1.0% (G) (%)	37.97	33.33	-12.2
FEV1.0% (T) (%)	35.29	32.21	-8.7
PEF (L/s)	2.85	2.89	1.4
V̇75 (L/s)	0.74	0.50	-32.4
V̇50 (L/s)	0.26	0.31	19.2
V̇25 (L/s)	0.16	0.17	6.3
V̇25/HT (L/s)	0.09	0.10	11.1
MMF (L/s)	0.24	0.26	8.3
CVI	0.41	0.39	-4.9
OI	8.03	9.73	21.2
IV50	2.48	3.09	24.6
V̇50/IV̇50	0.10	0.10	0.0
IV̇50/V̇50	9.54	9.97	4.5
	吸入前		
MVV (L/min)			
MVV/BSA (L/min/㎡)			
TV (L)			
RR (count/min)			

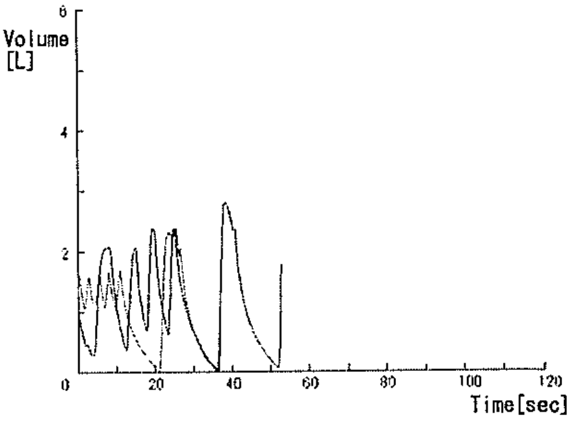

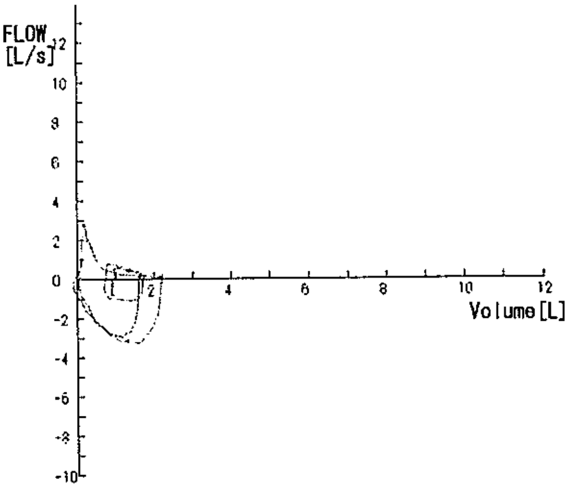

コメント.

薬効前、再検しましたが再現性とれず。

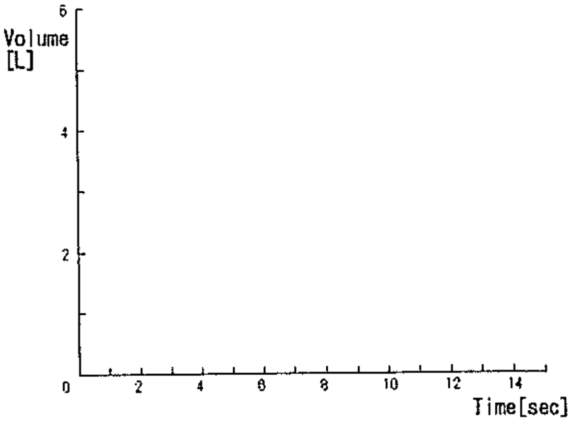

喫 煙：喫煙なし (BI=) 体表面積： 1.748 ㎡

No 5
No5
05

FRC		測定値	予測値	%予測値
FRC	(L)	5.40	3.86	139.9
RV	(L)	4.24	1.85	229.2
TLC	(L)	6.79	5.39	126.0
RV／TLC		62.44	39.12	159.6
VC	(L)	2.55	3.38	75.4
IRV	(L)	—		
TV	(L)	1.46		
ERV	(L)	1.16		
[illegible]		[illegible]	[illegible]	[illegible]
DLCO	(mL/min/mmHg)	15.46	16.04	96.4
DLCO'	(mL/min/mmHg)	12.30	17.30	71.1
RV (STPD)	(L)	3.52		
IVC (STPD)	(L)	2.16		
VA (STPD)	(L)	5.54		
VA' (STPD)	(L)	4.41		
DLCO/VA	(mL/min/mmHg/L)	2.79	4.26	65.5
B. H. T.	(s)	12.85		
FIHe	(%)	9.70		
FAHe	(%)	4.38		
FICO	(%)	0.289		
FACO	(%)	0.085		

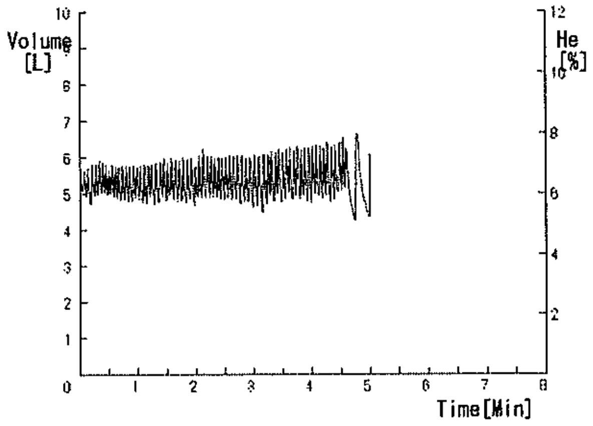

CV		測定値	予測値	%予測値
CV	(L)			
CC	(L)			
RV	(L)			
CV／VC	(%)			
CC／TLC	(%)			
⊿N_2	(%)			
MSAP				
ADS	(L)			

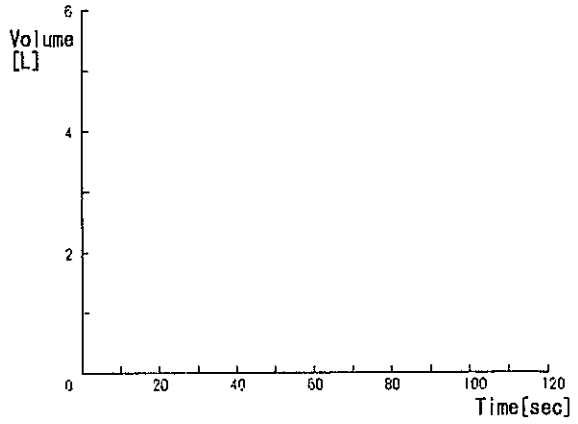

コメント.

薬効前、再検しましたが再現性とれず。

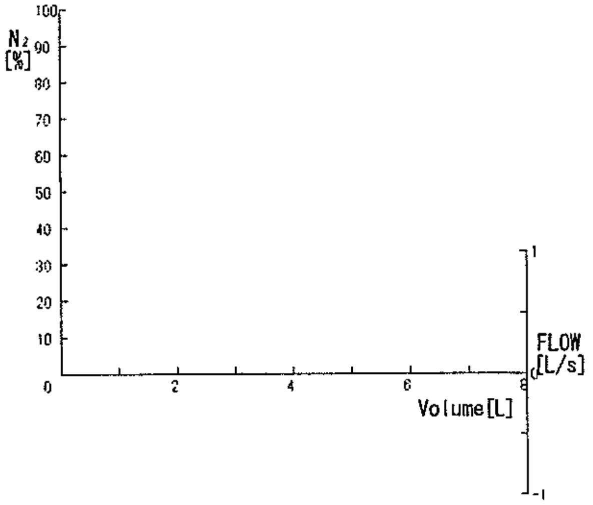

[illegible]

b6　　　　　　　　　　　　　　　　　　　　　　　　　　　　No6

スパイログラム	測定値	予測値	%予測値
肺活量　VC (L)	4.02	3.36	119.6
予備呼気量　ERV (L)	1.98	1.36	145.6
予備吸気量　IRV (L)	0.86		
努力性肺活量　FVC (L)	3.96	3.36	117.9
一秒量　FEV1.0 (L)	2.42	2.50	96.8
一秒率（G）FEV1.0%(%)	61.11	65.68	93.0
一秒率（T）FEV1.0%(%)	60.20		
最大中間呼気流量 (L/s)	0.98	2.58	38.0
ATI エアートラッピング指数	1.49		
公害指数	77.6		
最大換気量　MVV(L/min)			
分時換気量　MV (L/min)			
呼吸回数 (count/min)			
TV (L)			
フローボリューム曲線			
ピークフロー (L/s)	7.95	9.01	88.2
V̇75 (L/s)	4.74	7.16	66.2
V̇50 (L/s)	1.06	4.34	24.4
V̇25 (L/s)	0.41	1.10	37.3
V̇50/V̇25	2.59		
V̇25/HT (L/s/m)	0.24	0.94	25.5
肺気量			
機能的残気量　FRC (L)			
全肺気量　TLC (L)			
残気量　RV (L)			
肺活量　VC (L)			
残気率　RV/TLC (%)			
肺拡散能力			
DLCO (mL/min/mmHg)			
VA (STPD) (L)			
DLCO/VA(mL/min/mmHg/L)			
B.H.TIME (s)			

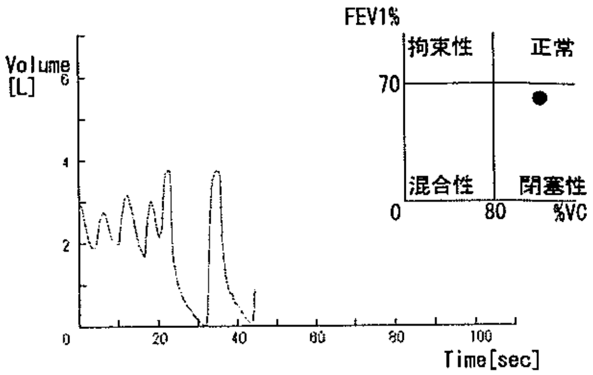

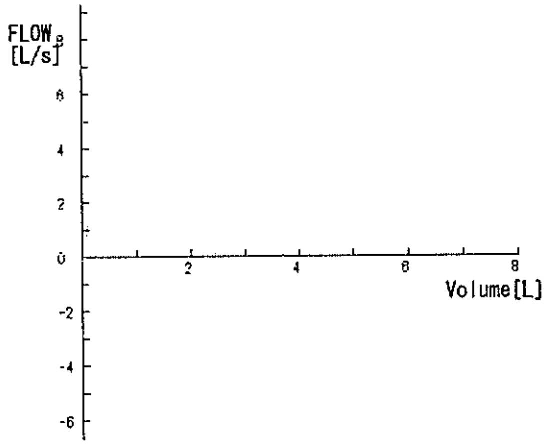

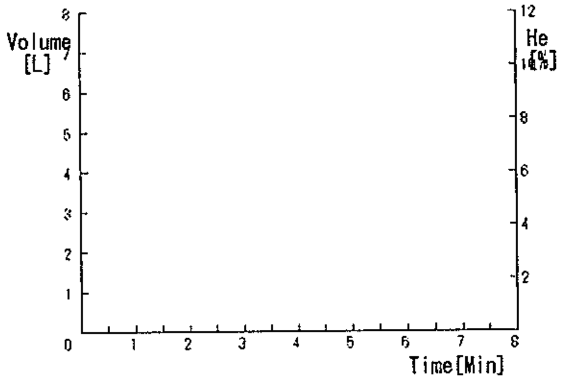

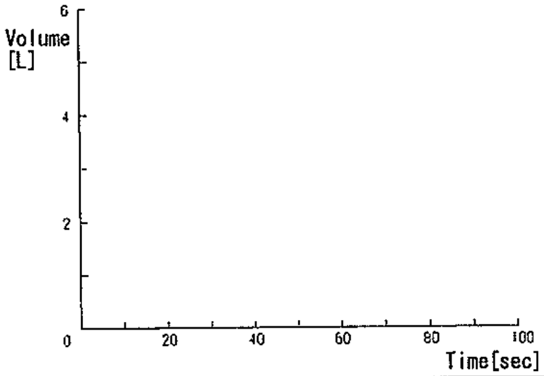

コメント.

107

[illegible]

No7

スパイログラム	測定値	予測値	%予測値
肺活量 VC (L)	2.50	3.08	81.2
予備呼気量 ERV (L)	0.06	1.25	4.8
予備吸気量 IRV (L)	1.35		
努力性肺活量 FVC (L)	2.46	3.08	79.9
一秒量 FEV1.0 (L)	1.56	2.02	77.2
一秒率（G） FEV1.0%(%)	63.41	65.68	96.5
一秒率（T） FEV1.0%(%)	62.40		
最大中間呼気流量 (L/s)	0.42	2.29	18.3
ATI エアートラッピング指数	1.60		
公害指数	54.5		
フローボリューム曲線			
ピークフロー (L/s)	7.30	8.65	84.4
V̇75 (L/s)	5.71	6.67	85.6
V̇50 (L/s)	0.95	3.98	23.9
V̇25 (L/s)	0.11	0.90	12.2
V̇50／V̇25	8.64		
V̇25／HT (L/s/m)	0.07	0.94	7.4
IV50 (L/s)	3.20		
V̇50／IV̇50	0.30		
IV̇50／V̇50	3.37		
肺気量			
機能的残気量 FRC (L)			
全肺気量 TLC (L)			
残気量 RV (L)			
肺活量 VC (L)			
残気率 RV／TLC (%)			
肺拡散能力			
DLCO (mL/min/mmHg)			
VA (STPD) (L)			
DLCO/VA (mL/min/mmHg/L)			
B. H. TIME (s)			

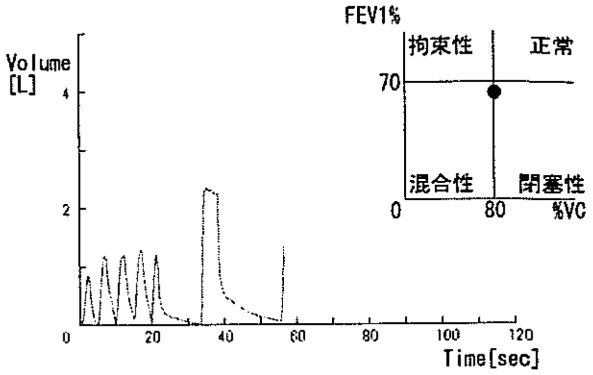

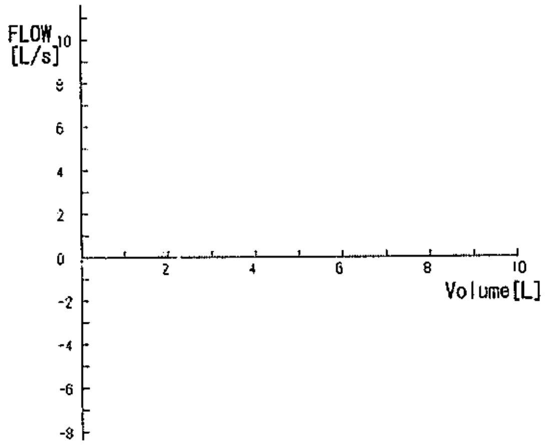

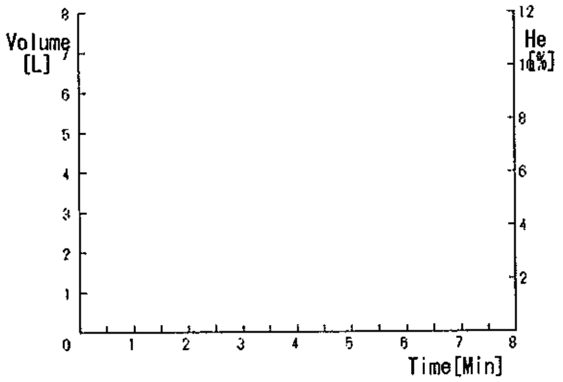

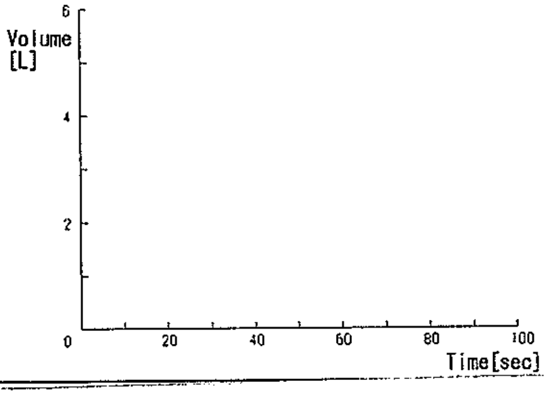

コメント.

喫 煙: (BI=) 体表面積: [illegible]

08

No8

スパイログラム	測定値	予測値	%予測値
肺活量 VC (L)	2.05	3.10	66.1
予備呼気量 ERV (L)	0.53	1.25	42.4
予備吸気量 IRV (L)	0.62		
努力性肺活量 FVC (L)	1.88	3.10	60.6
一秒量 FEV1.0 (L)	0.98	2.06	47.6
一秒率（G） FEV1.0%(%)	52.13	65.68	79.4
一秒率（T） FEV1.0%(%)	47.80		
最大中間呼気流量 (L/s)	0.27	2.36	11.4
ATI エアートラッピング指数	8.29		
公害指数	33.8		
フローボリューム曲線			
ピークフロー (L/s)	3.59	8.68	41.4
$\dot{V}$75 (L/s)	1.38	6.70	20.6
$\dot{V}$50 (L/s)	0.36	4.00	9.0
$\dot{V}$25 (L/s)	0.11	0.91	12.1
$\dot{V}$50/$\dot{V}$25	3.27		
$\dot{V}$25/HT (L/s/m)	0.07	0.95	7.4
I$\dot{V}$50 (L/s)	—		
$\dot{V}$50/I$\dot{V}$50	—		
I$\dot{V}$50/$\dot{V}$50	—		
肺気量			
機能的残気量 FRC (L)			
全肺気量 TLC (L)			
残気量 RV (L)			
肺活量 VC (L)			
残気率 RV/TLC (%)			
肺拡散能力			
DLCO (mL/min/mmHg)			
VA (STPD) (L)			
DLCO/VA (mL/min/mmHg/L)			
B.H.TIME (s)			

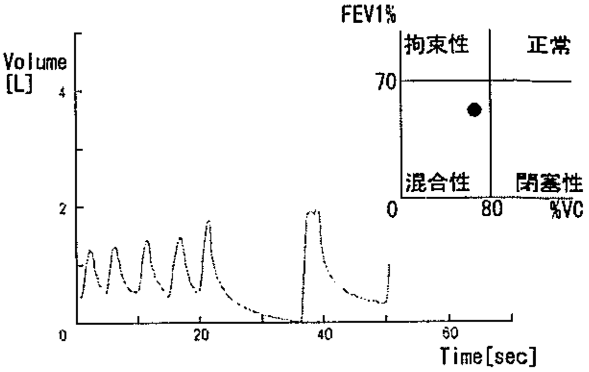

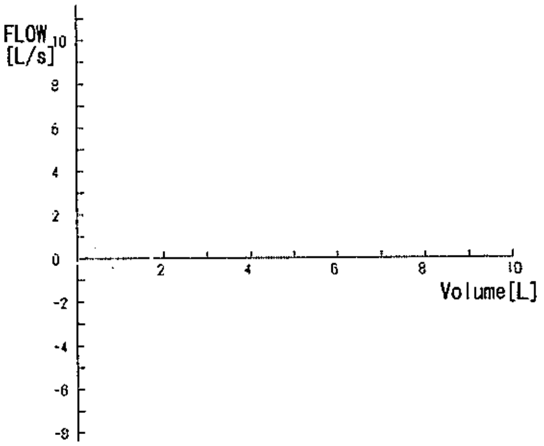

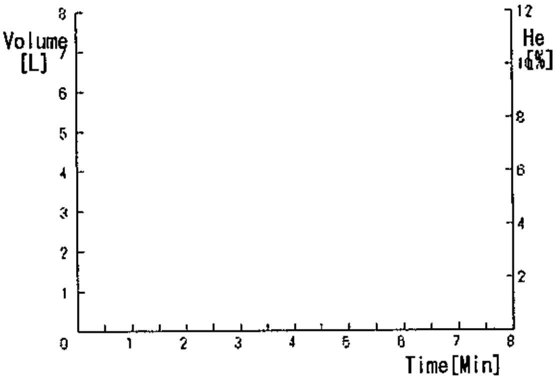

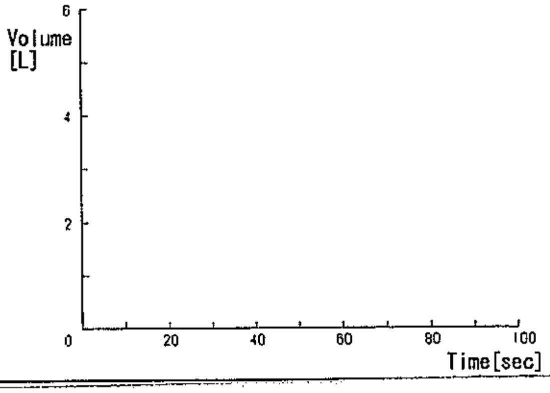

コメント.

喫 歴：喫煙なし (BI=) 体表面積：[illegible]

109

No9

スパイログラム	測定値	予測値	%予測値
肺活量 VC (L)	3.53	3.32	106.3
予備呼気量 ERV (L)	0.84	1.41	59.6
予備吸気量 IRV (L)	1.22		
努力性肺活量 FVC (L)	3.21	3.32	96.7
一秒量 FEV1.0 (L)	1.59	2.47	64.4
一秒率（G） FEV1.0%(%)	49.53	66.05	75.0
一秒率（T） FEV1.0%(%)	45.04		
最大中間呼気流量 (L/s)	0.45	3.13	14.4
ATI エアートラッピング指数	9.07		
公害指数	47.9		
フローボリューム曲線			
ピークフロー (L/s)	5.29	9.02	58.6
V̇75 (L/s)	1.51	7.29	20.7
V̇50 (L/s)	0.67	4.59	14.6
V̇25 (L/s)	0.16	1.48	10.8
V̇50／V̇25	4.19		
V̇25／HT (L/s/m)	0.10	1.08	9.3
IV50 (L/s)	4.14		
V̇50／IV̇50	0.16		
IV̇50／V̇50	6.18		
肺気量			
機能的残気量 FRC (L)			
全肺気量 TLC (L)			
残気量 RV (L)			
肺活量 VC (L)			
残気率 RV／TLC (%)			
肺拡散能力			
DLCO (mL/min/mmHg)			
VA (STPD) (L)			
DLCO/VA (mL/min/mmHg/L)			
B.H.TIME (s)			

コメント.

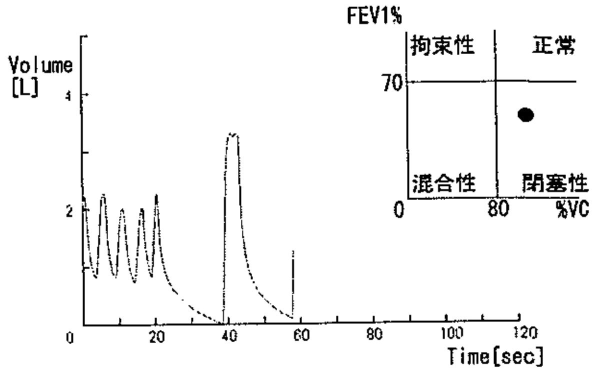

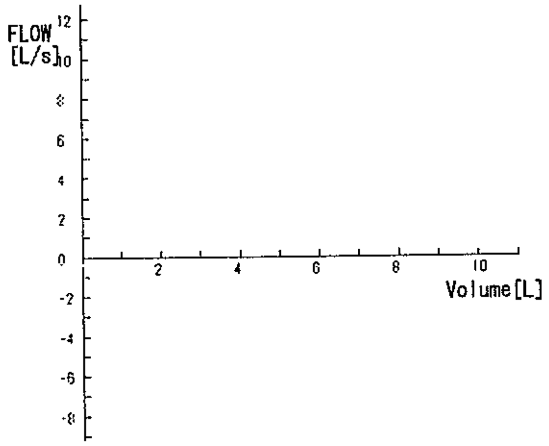

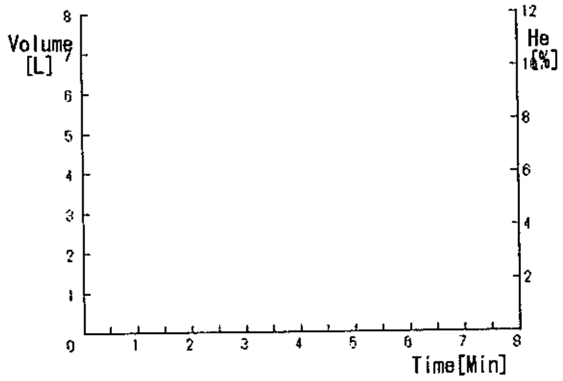

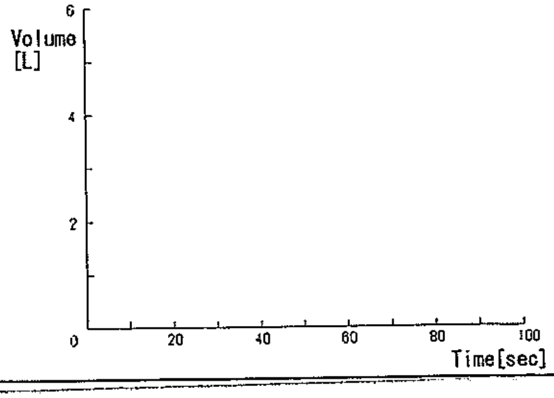

09

喫　煙：喫煙なし　（BI=　　）　体表面積：　1.765　㎡

No9

SVC		吸入前	吸入後	改善率
VC	(L)	3.53	3.67	4.0
ERV	(L)	0.84	0.93	10.7
IRV	(L)	1.22	1.06	-13.1
TV	(L)	1.47	1.68	14.3
IC	(L)	2.69	2.74	1.9

		吸入前		
FVC	(L)	3.21	3.70	15.3
FEV1.0	(L)	1.59	1.83	15.1
FEV1.0% (G)	(%)	49.53	49.46	-0.1
FEV1.0% (T)	(%)	45.04	49.86	10.7
PEF	(L/s)	5.29	6.38	20.6
V̇75	(L/s)	1.51	1.66	9.9
V̇50	(L/s)	0.67	0.79	17.9
V̇25	(L/s)	0.16	0.15	-6.3
V̇25/HT	(L/s)	0.10	0.09	-10.0
MMF	(L/s)	0.45	0.46	2.2
CVI		0.50	0.69	38.0
OI		8.33	9.21	10.6
IV50		4.14	2.97	-28.3
V̇50/IV̇50		0.16	0.27	68.8
IV̇50/V̇50		6.18	3.76	-39.2

		吸入前		
MVV	(L/min)			
MVV/BSA	(L/min/㎡)			
TV	(L)			
RR	(count/min)			

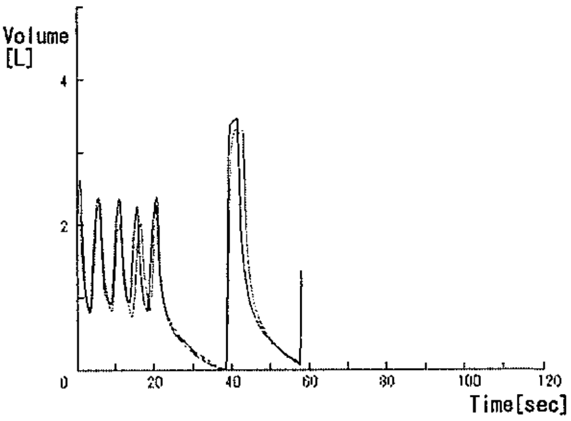

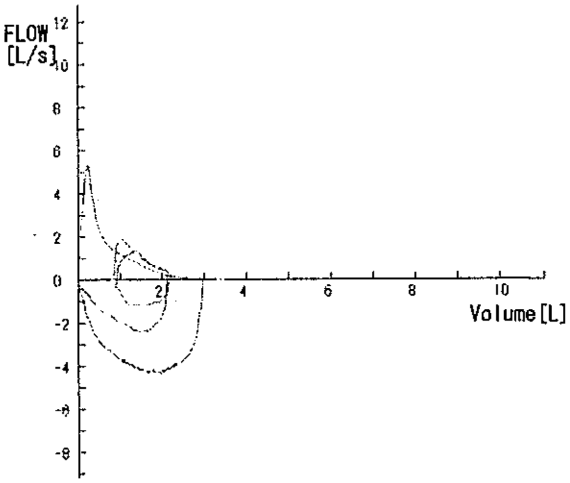

コメント.

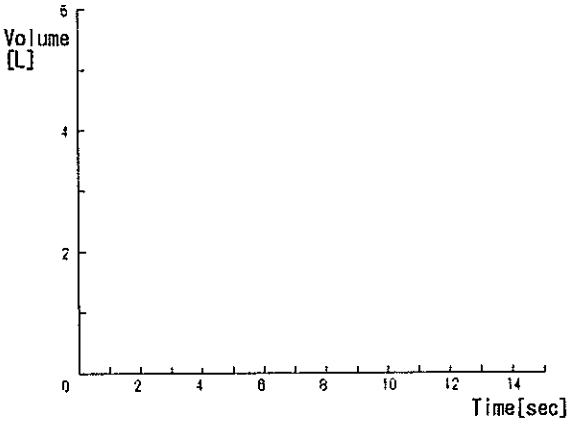

喫　煙：喫煙なし　(BI=　　)　体表面積：　[illegible]

510　　　　　　　　　　　　　　　　　　　　　　　　　　No10

スパイログラム	測定値	予測値	%予測値
肺活量　VC (L)	3.01	3.38	89.1
予備呼気量　ERV (L)	0.91	1.37	66.4
予備吸気量　IRV (L)	0.92		
努力性肺活量　FVC (L)	2.99	3.38	88.5
一秒量　FEV1.0 (L)	1.66	2.53	65.6
一秒率（G）FEV1.0% (%)	55.52	65.68	84.5
一秒率（T）FEV1.0% (%)	55.15		
最大中間呼気流量 (L/s)	0.49	2.77	17.7
ATI　エアートラッピング指数	0.66		
公害指数	51.7		
フローボリューム曲線			
ピークフロー (L/s)	5.19	9.02	57.5
V̇75 (L/s)	2.29	7.21	31.8
V̇50 (L/s)	0.60	4.38	13.7
V̇25 (L/s)	0.19	1.15	16.5
V̇50/V̇25	3.16		
V̇25/HT (L/s/m)	0.11	0.98	11.2
IV50 (L/s)	1.50		
V̇50/IV̇50	0.40		
IV̇50/V̇50	2.50		
肺気量			
機能的残気量　FRC (L)			
全肺気量　TLC (L)			
残気量　RV (L)			
肺活量　VC (L)			
残気率　RV/TLC (%)			
肺拡散能力			
DLCO (mL/min/mmHg)			
VA (STPD) (L)			
DLCO/VA (mL/min/mmHg/L)			
B.H.TIME (s)			

コメント.

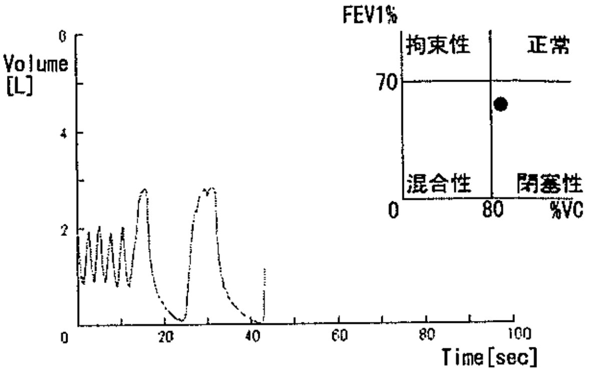

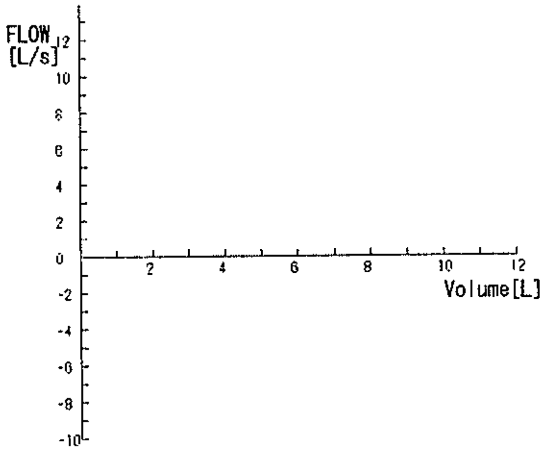

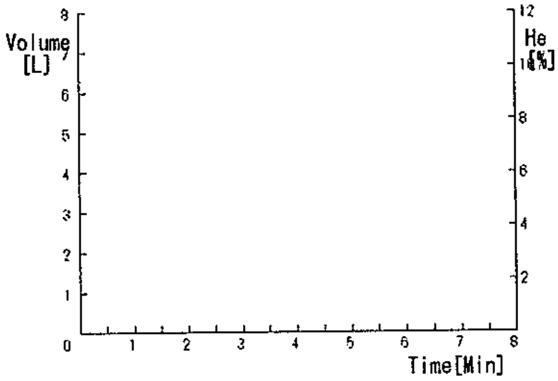

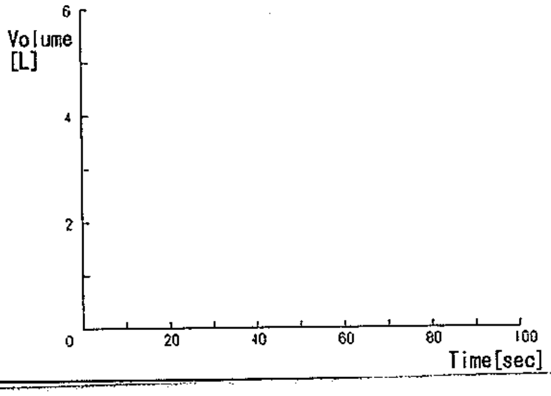

喫　煙：喫煙なし　(BI=　　)　体表面積：　1. 6 5 0 m

1010　　　　　　　　No 10

SVC	吸入前	吸入後	改善率
VC (L)	3.01	3.42	13.6
ERV (L)	0.91	1.30	42.9
IRV (L)	0.92	1.14	23.9
TV (L)	1.18	0.98	-17.0
IC (L)	2.10	2.12	1.0

	吸入前		
FVC (L)	2.99	3.27	9.4
FEV1.0 (L)	1.66	1.88	13.3
FEV1.0% (G) (%)	55.52	57.49	3.5
FEV1.0% (T) (%)	55.15	54.97	-0.3
PEF (L/s)	5.19	6.85	32.0
$\dot{V}$75 (L/s)	2.29	2.78	21.4
$\dot{V}$50 (L/s)	0.60	0.88	46.7
$\dot{V}$25 (L/s)	0.19	0.27	42.1
$\dot{V}$25/HT (L/s)	0.11	0.16	45.5
MMF (L/s)	0.49	0.67	36.7
CVI	1.36	0.89	-34.6
OI	5.38	5.88	9.3
IV50	1.50	2.61	74.0
$\dot{V}$50/I$\dot{V}$50	0.40	0.34	-15.0
I$\dot{V}$50/$\dot{V}$50	2.50	2.97	18.8

	吸入前		
MVV (L/min)			
MVV/BSA (L/min/m²)			
TV (L)			
RR (count/min)			

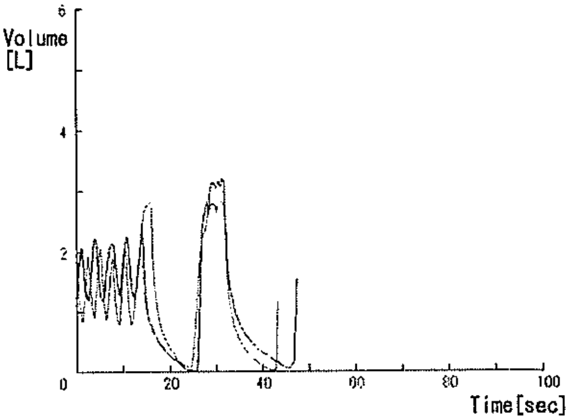

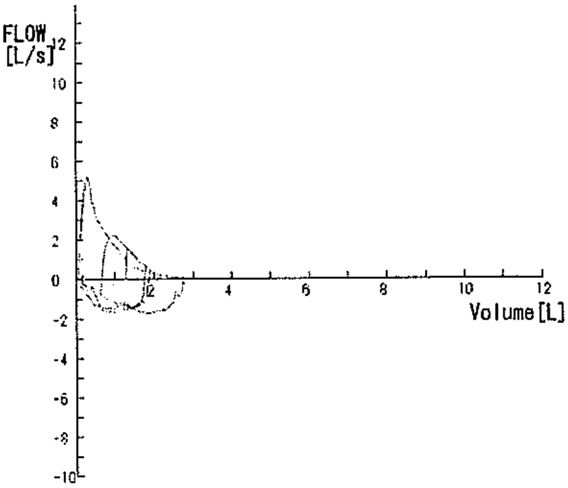

コメント.

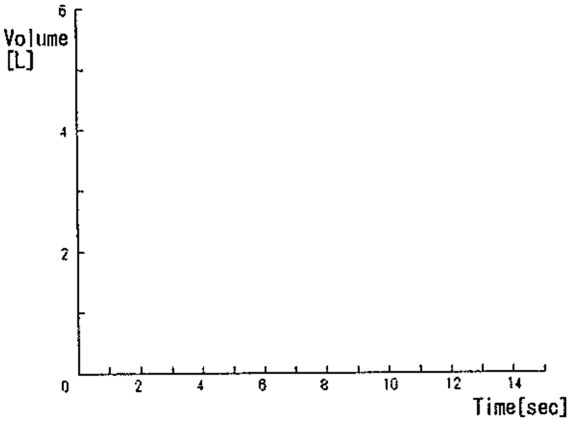

No11

喫　煙：　　　　(BI=　　)　体表面積：　1.675 ㎡

スパイログラム	測定値	予測値	%予測値
肺活量　VC (L)	3.32	3.50	94.9
予備呼気量　ERV (L)	1.16	1.44	80.6
予備吸気量　IRV (L)	1.35		
努力性肺活量　FVC (L)	3.37	3.50	96.3
一秒量　FEV1.0 (L)	1.19	2.74	43.4
一秒率（G）FEV1.0%(%)	35.31	65.68	53.8
一秒率（T）FEV1.0%(%)	35.84		
最大中間呼気流量 (L/s)	0.37	2.99	12.4
ATI エアートラッピング指数	-1.51		
公害指数	35.4		
フローボリューム曲線			
ピークフロー (L/s)	3.40	9.17	37.1
V̇75 (L/s)	0.81	7.47	10.8
V̇50 (L/s)	0.39	4.61	8.5
V̇25 (L/s)	0.20	1.32	15.2
V̇50/V̇25	1.95		
V̇25/HT (L/s/m)	0.11	1.01	10.9
IV50 (L/s)	3.99		
V̇50/IV̇50	0.10		
IV̇50/V̇50	10.23		
肺気量			
機能的残気量　FRC (L)			
全肺気量　TLC (L)			
残気量　RV (L)			
肺活量　VC (L)			
残気率　RV/TLC (%)			
肺拡散能力			
DLCO (mL/min/mmHg)			
VA (STPD) (L)			
DLCO VA (mL/min/mmHg/L)			
B.H.TIME (s)			

コメント.

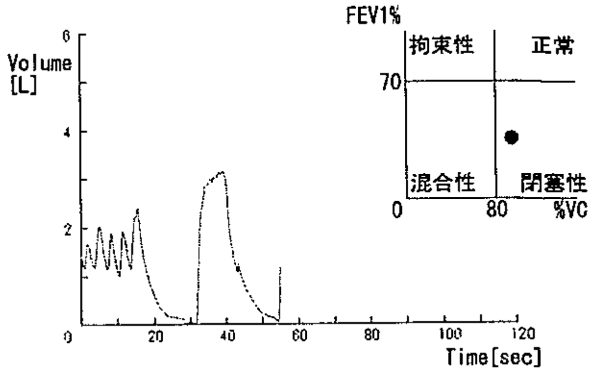

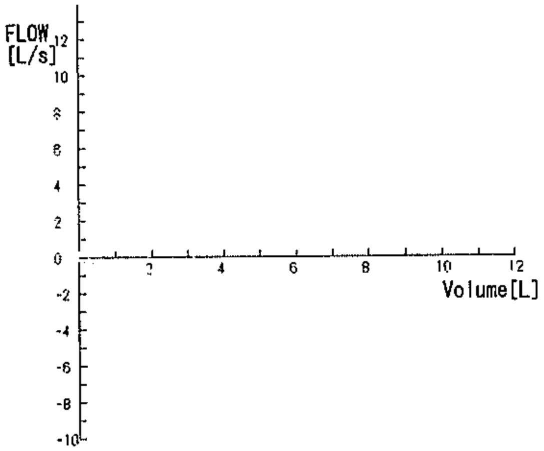

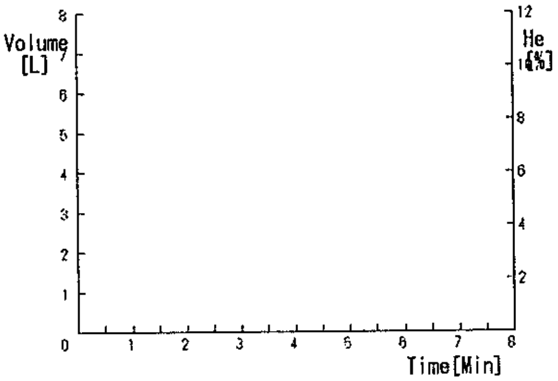

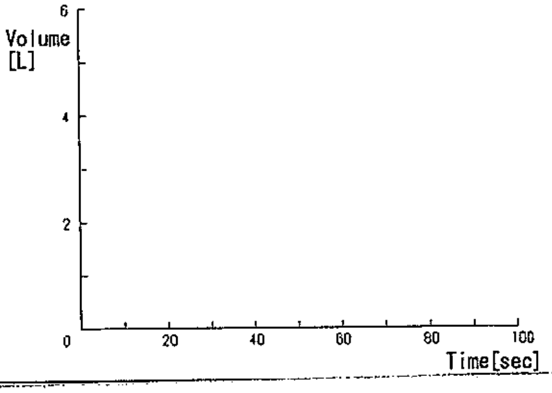

No 11

[illegible] (BT=) 体表面積： 1.675 m

スパイログラム	測定値	予測値	%予測値
肺活量 VC (L)	3.32	3.50	94.9
予備呼気量 ERV (L)	1.16	1.44	80.6
予備吸気量 IRV (L)	1.35		
努力性肺活量 FVC (L)	3.37	3.50	96.3
一秒量 FEV1.0 (L)	1.19	2.74	43.4
一秒率（G） FEV1.0%(%)	35.31	65.68	53.8
一秒率（T） FEV1.0%(%)	35.84		
最大中間呼気流量 (L/s)	0.37	2.99	12.4
ATI エアートラッピング指数	-1.51		
公害指数	35.4		
フローボリューム曲線			
ピークフロー (L/s)	3.40	9.17	37.1
V̇75 (L/s)	0.81	7.47	10.8
V̇50 (L/s)	0.39	4.61	8.5
V̇25 (L/s)	0.20	1.32	15.2
V̇50/V̇25	1.95		
V̇25/HT (L/s/m)	0.11	1.01	10.9
IV50 (L/s)	3.99		
V̇50/IV̇50	0.10		
IV̇50/V̇50	10.23		
肺気量			
機能的残気量 FRC (L)			
全肺気量 TLC (L)			
残気量 RV (L)			
肺活量 VC (L)			
残気率 RV/TLC (%)			
肺拡散能力			
DLCO (mL/min/mmHg)			
VA (STPD) (L)			
DLCO/VA (mL/min/mmHg/L)			
B.H.TIME (s)			

コメント.

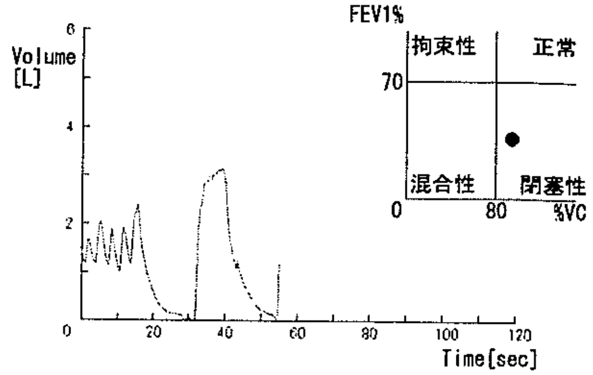

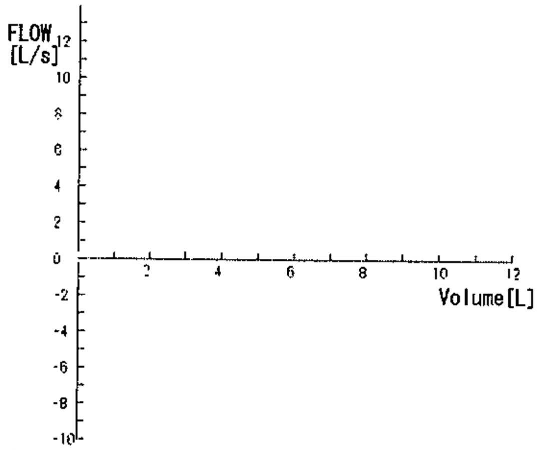

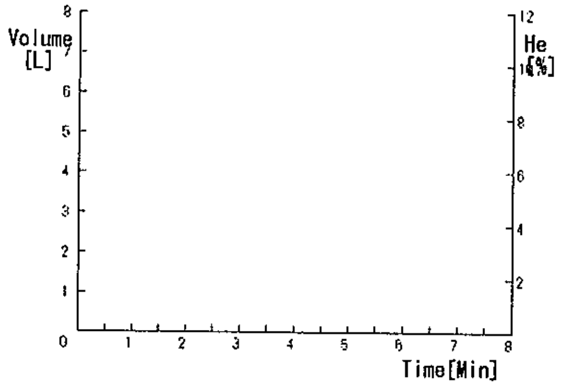

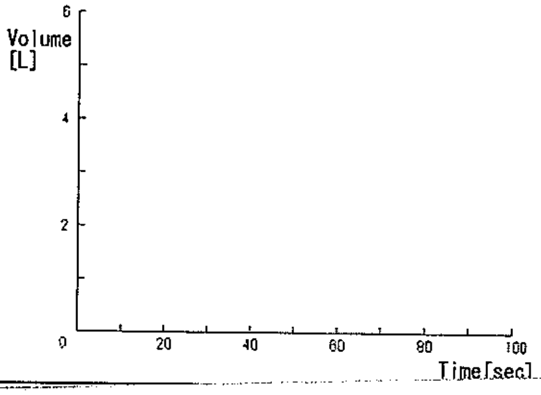

No 11

Vo12

喫 煙： (BI=) 体表面積： 1.753 m

No12

スパイログラム	測定値	予測値	%予測値
肺活量 VC (L)	3.41	3.18	107.2
予備呼気量 ERV (L)	1.27	1.33	95.5
予備吸気量 IRV (L)	1.08		
努力性肺活量 FVC (L)	3.19	3.18	100.3
一秒量 FEV1.0 (L)	1.20	2.19	54.8
一秒率（G） FEV1.0%(%)	37.62	65.68	57.3
一秒率（T） FEV1.0%(%)	35.19		
最大中間呼気流量 (L/s)	0.25	2.85	8.8
ATI エアートラッピング指数	6.45		
公害指数	38.3		
フローボリューム曲線			
ピークフロー (L/s)	4.69	8.78	53.4
V̇75 (L/s)	0.89	6.98	12.8
V̇50 (L/s)	0.28	4.32	6.5
V̇25 (L/s)	0.13	1.26	10.3
V̇50/V̇25	2.15		
V̇25/HT (L/s/m)	0.08	1.05	7.6
IV50 (L/s)	3.33		
V̇50/IV̇50	0.08		
IV̇50/V̇50	11.89		
肺気量			
機能的残気量 FRC (L)			
全肺気量 TLC (L)			
残気量 RV (L)			
肺活量 VC (L)			
残気率 RV/TLC (%)			
肺拡散能力			
DLCO (mL/min/mmHg)			
VA (STPD) (L)			
DLCO/VA (mL/min/mmHg/L)			
B.H.TIME (s)			

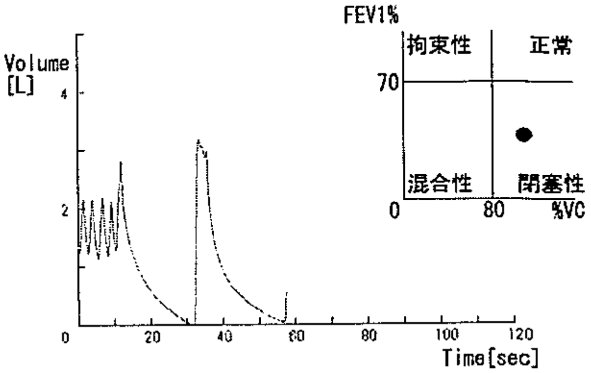

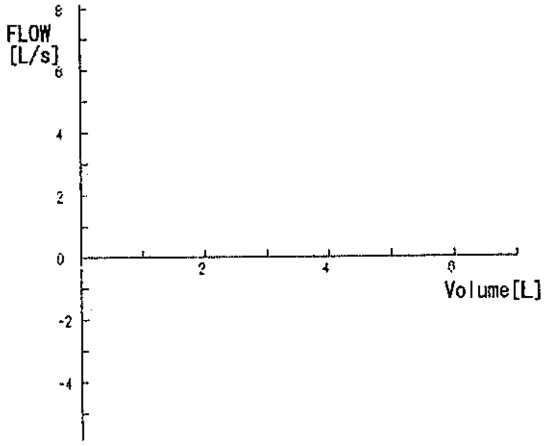

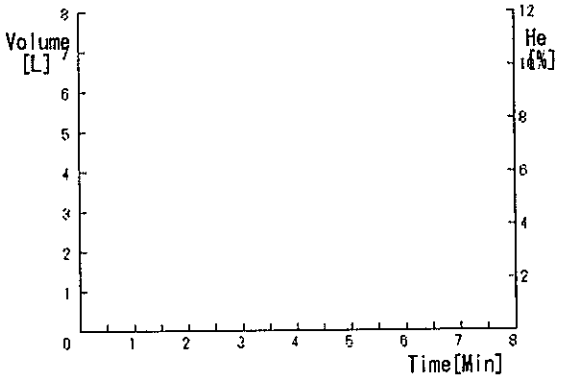

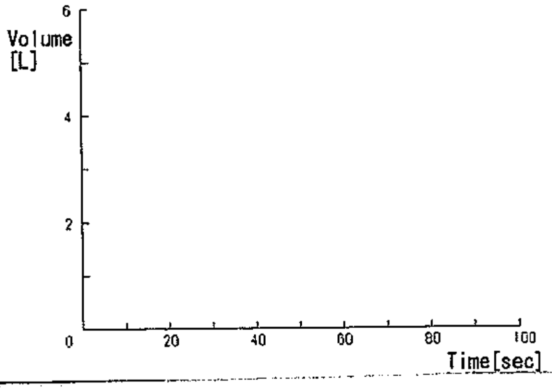

コメント.

再検済み

No13

喫　煙：　　　　　(BI=　　　)　　体表面積：　1．663 m

スパイログラム	測定値	予測値	%予測値
肺活量　VC (L)	3.81	3.16	120.6
予備呼気量　ERV (L)	0.88	1.28	68.8
予備吸気量　IRV (L)	1.26		
努力性肺活量　FVC (L)	3.68	3.16	116.5
一秒量　FEV1.0 (L)	1.37	2.16	63.4
一秒率（G）　FEV1.0%(%)	37.23	65.68	56.7
一秒率（T）　FEV1.0%(%)	35.96		
最大中間呼気流量 (L/s)	0.39	2.33	16.7
ATI　エアートラッピング指数	3.41		
公害指数	46.9		
フローボリューム曲線			
ピークフロー (L/s)	5.24	8.76	59.8
V̇75 (L/s)	1.04	6.81	15.3
V̇50 (L/s)	0.45	4.08	11.0
V̇25 (L/s)	0.19	0.95	20.0
V̇50/V̇25	2.37		
V̇25/HT (L/s/m)	0.12	0.93	12.9
IV50 (L/s)	1.14		
V̇50/IV̇50	0.39		
IV̇50/V̇50	2.53		
肺気量			
機能的残気量　FRC (L)			
全肺気量　TLC (L)			
残気量　RV (L)			
肺活量　VC (L)			
残気率　RV/TLC (%)			
肺拡散能力			
DLCO (mL/min/mmHg)			
VA (STPD) (L)			
DLCO/VA (mL/min/mmHg/L)			
B.H.TIME (s)			

コメント.

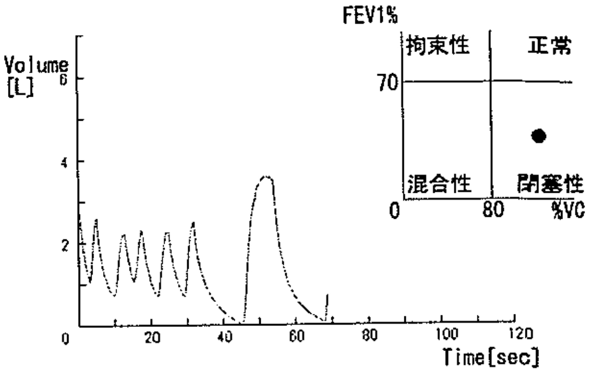

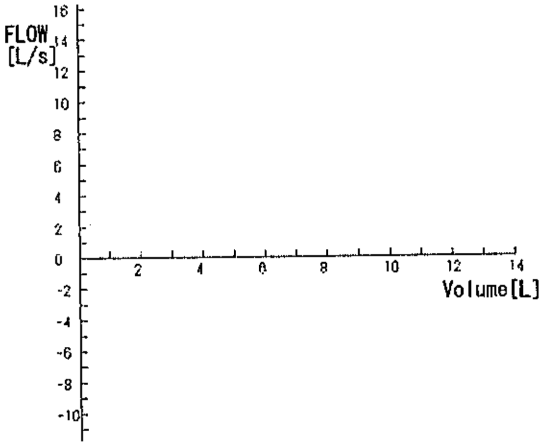

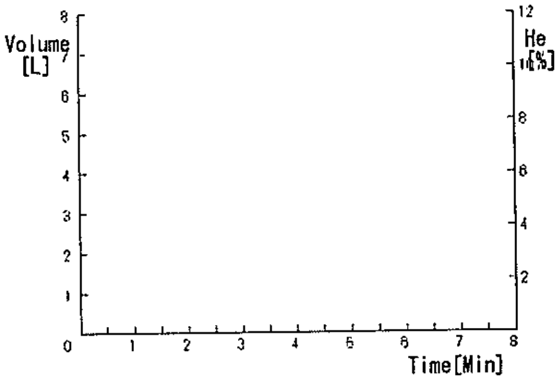

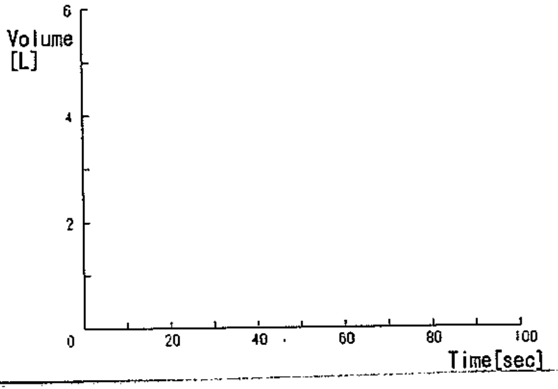

No13

ω14

No14

喫 煙： (BI=) 体表面積： 1.521 m

スパイログラム	測定値	予測値	%予測値
肺活量 VC (L)	3.31	2.84	116.5
予備呼気量 ERV (L)	1.59	1.07	148.6
予備吸気量 IRV (L)	1.05		
努力性肺活量 FVC (L)	3.29	2.84	115.8
一秒量 FEV1.0 (L)	2.20	2.65	83.0
一秒率（G） FEV1.0%(%)	66.87	79.32	84.3
一秒率（T） FEV1.0%(%)	66.47		
最大中間呼気流量 (L/s)	1.23	3.29	37.4
ATI エアートラッピング指数	0.60		
公害指数	77.5		
フローボリューム曲線			
ピークフロー (L/s)	3.85	7.87	48.9
V̇75 (L/s)	3.44	5.78	59.5
V̇50 (L/s)	1.52	4.42	34.4
V̇25 (L/s)	0.51	2.08	24.5
V̇50/V̇25	2.98		
V̇25/HT (L/s/m)	0.30	1.09	27.5
IV50 (L/s)	—		
V̇50/IV̇50	—		
IV̇50/V̇50	—		
肺気量			
機能的残気量 FRC (L)			
全肺気量 TLC (L)			
残気量 RV (L)			
肺活量 VC (L)			
残気率 RV/TLC (%)			
肺拡散能力			
DLCO (mL/min/mmHg)			
VA (STPD) (L)			
DLCO/VA (mL/min/mmHg/L)			
B. H. TIME (s)			

コメント.

吸気肺活量うまく行えず。

複数回測定しました。

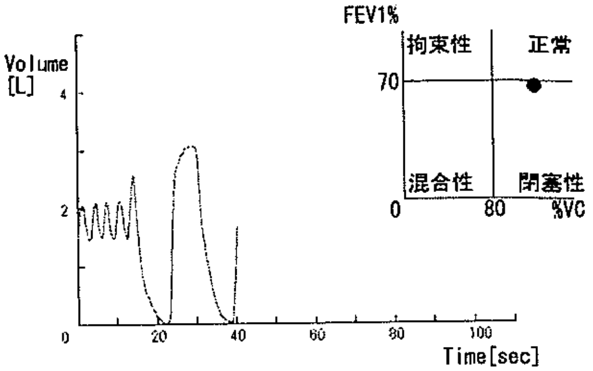

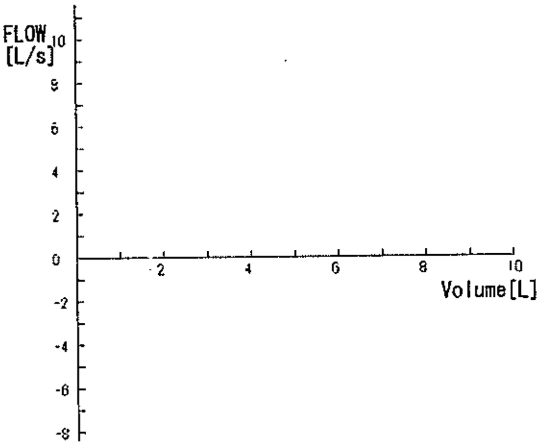

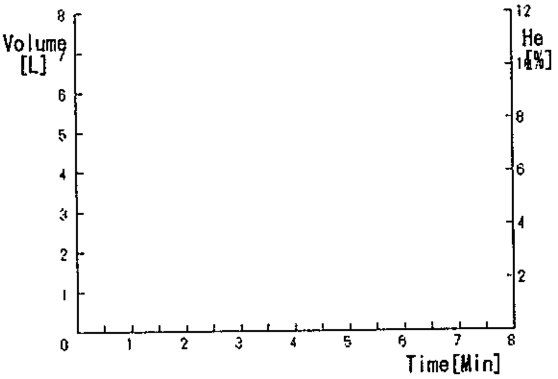

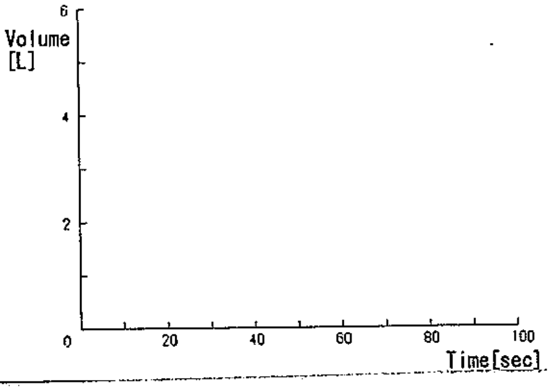

015

No15

喫　煙：　(BI=　　)　体表面積：　1.667 m

スパイログラム		測定値	予測値	%予測値
肺活量	VC (L)	2.76	3.30	83.6
予備呼気量	ERV (L)	0.47	1.40	33.6
予備吸気量	IRV (L)	1.49		
努力性肺活量	FVC (L)	2.65	3.30	80.3
一秒量	FEV1.0 (L)	1.82	2.43	74.9
一秒率（G）	FEV1.0%(%)	68.68	67.17	102.2
一秒率（T）	FEV1.0%(%)	65.94		
最大中間呼気流量	(L/s)	0.92	3.19	28.8
ATI	エアートラッピング指数	3.99		
公害指数		55.2		
フローボリューム曲線				
ピークフロー	(L/s)	7.08	9.12	77.6
V̇75	(L/s)	4.39	7.21	60.9
V̇50	(L/s)	1.35	4.58	29.5
V̇25	(L/s)	0.31	1.55	20.0
V̇50／V̇25		4.35		
V̇25／HT	(L/s/m)	0.19	1.11	17.1
IV50	(L/s)	1.76		
V̇50／IV̇50		0.77		
IV̇50／V̇50		1.30		
肺気量				
機能的残気量	FRC (L)			
全肺気量	TLC (L)			
残気量	RV (L)			
肺活量	VC (L)			
残気率	RV／TLC (%)			
肺拡散能力				
DLCO	(mL/min/mmHg)			
VA (STPD)	(L)			
DLCO/VA	(mL/min/mmHg/L)			
B. H. TIME	(s)			

コメント.

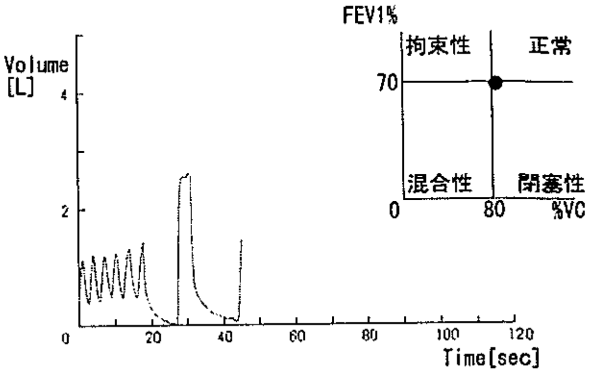

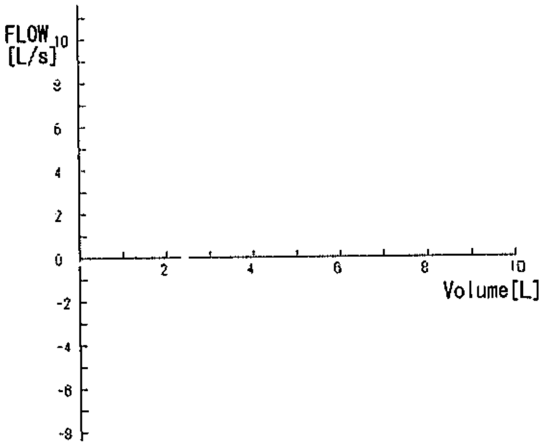

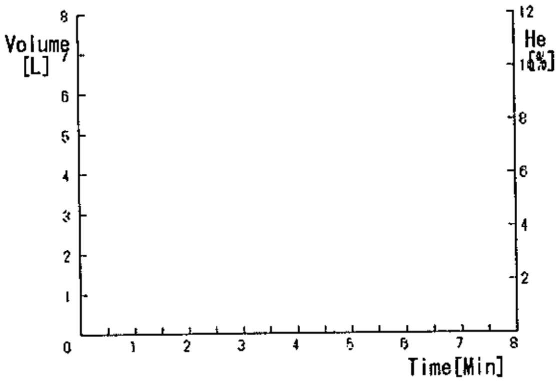

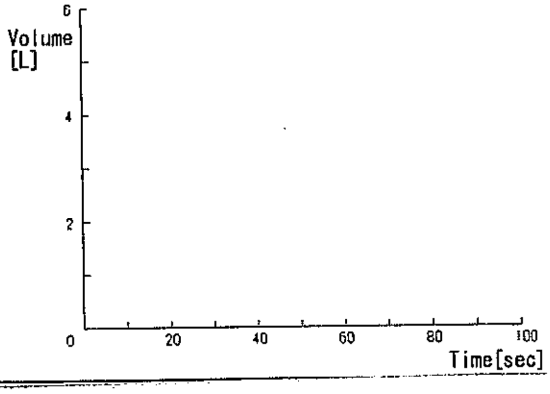

Supplement: S1 Dataset — (ZIP) [file pone.0276859.s009.zip › PLOS_ONE_Æ╟ë┴âfü[â^/spirographdataCOPD No1toNo15.pdf]
